# Estimating the Attack Ratio of Dengue Epidemics under Time-varying Force of Infection using Aggregated Notification Data

**DOI:** 10.1038/srep18455

**Published:** 2015-12-17

**Authors:** Flavio Codeço Coelho, Luiz Max de Carvalho

**Affiliations:** 1 Escola de Matemática Aplicada, Fundação Getulio Vargas (FGV), Rio de Janeiro – RJ, Brazil; 2 Programa de Computação Científica (PROCC), Fundação Oswaldo Cruz, Rio de Janeiro – RJ, Brazil

## Abstract

Quantifying the attack ratio of disease is key to epidemiological inference and public health planning. For multi-serotype pathogens, however, different levels of serotype-specific immunity make it difficult to assess the population at risk. In this paper we propose a Bayesian method for estimation of the attack ratio of an epidemic and the initial fraction of susceptibles using aggregated incidence data. We derive the probability distribution of the effective reproductive number, *R*_*t*_, and use MCMC to obtain posterior distributions of the parameters of a single-strain SIR transmission model with time-varying force of infection. Our method is showcased in a data set consisting of 18 years of dengue incidence in the city of Rio de Janeiro, Brazil. We demonstrate that it is possible to learn about the initial fraction of susceptibles and the attack ratio even in the absence of serotype specific data. On the other hand, the information provided by this approach is limited, stressing the need for detailed serological surveys to characterise the distribution of serotype-specific immunity in the population.

Dengue is an arthropod-borne febrile disease caused by a flavivirus with four serotypes (DEN-1, 2, 3 and 4) which causes an estimated 50 million infections each year^1^. In humans, immunity against a particular serotype is considered permanent after the exposure and cross immunity to other serotypes is considered short lived^2^ although some studies argue for a longer duration of cross-immunity^3^,^4^. As a consequence, the proportions of viral serotypes co-circulating at any point in time are strongly dependent on previous incidence patterns of the disease, which determine the number of individuals susceptible to each serotype at any point in time.

Dengue transmission is also modulated by environmental conditions, among which, temperature, due to its effects on the vector reproduction, stands out as a strong predictor of incidence^5^,^6^. In places with sufficient seasonal temperature variation, dengue is predominantly a summer disease. So it is fair to say that these environmental fluctuations play a key role in determining beginning and end of epidemic periods. This climatic influence is exerted mainly through its effects on the force of infection, which cannot be taken as constant^7^ but rather as a seasonal (oscillating) function of time. The long term dynamics of dengue is also modulated by the alternation of virus types in circulation. Demographics also plays a role in replenishing the population of susceptibles.

The attack ratio (AR) of a disease is a measure of morbidity defined as the number of new cases divided by the population at risk. For dengue epidemics, it can be difficult to calculate the AR due to the lack of knowledge of the population at risk. The population at risk in this case is the number of susceptibles to the circulating virus type(s) before a given epidemic. Thus, in order to calculate the attack ratio, we need to determine the number of susceptibles to the circulating virus types right before the epidemic, which is virtually impossible without regular virological surveys.

The attack ratio is also influenced by the reproductive number of the disease^8^,^9^, which is closely associated with the force of infection. Thus the incorporation of the effective reproduction number, *R*_*t*_, as a function of time, is crucial to an accurate estimation of the AR of seasonal diseases like dengue and Influenza.

Other methods for estimating the number of susceptibles while accommodating time-varying force of infection have been proposed before, for measles^10^,^11^, a disease that shows remarkable seasonality. These methods try to reconstruct the entire series of infectious and susceptibles from case data using deterministic models and generally work well for measles because there is a one-to-one relationship between exposure and immunity, since measles is caused by a single-strain pathogen. Recently, methods in the same fashion were developed for dengue when serotype-specific data is available^12^. When such data is not available, the series of susceptibles to all possible serotypes, cannot be reconstructed based solely on a deterministic transmission model, since the arrival/re-emergence of new serotypes, an intrinsically stochastic event, can drastically change the pool of susceptibles, throwing off any sequential estimation based on the incidence dynamics.

Dengue has been reintroduced in Rio de Janeiro in 1986 after being absent for 68 years^13^. During the last decades of the 20th century only DEN-1 and DEN-2 serotypes were in circulation. The remaining serotypes DEN-3 and DEN-4, arrived respectively in 2000 and 2011^14^,^15^. Due to this patterns of recent re-introduction of the disease, its incidence dynamics is still dominated by the introduction events and environmental determinants of transmission.

In this paper, we propose a new approach to estimate the number (fraction) of susceptibles using a simplified model of dengue transmission based on a single-strain Susceptible-Infectious-Removed (SIR) model with time-varying infection rate. In order to bypass the limitations of not knowing the serotype-specific seroprevalence and the exact behaviour of the force of infection through time, we propose to inform the time-varying transmissibility using the *R*_*t*_ series derived from the notification data^16^. We extend a Bayesian framework previously used to estimate the number of susceptibles in Influenza epidemics in Europe^17^ to include time-varying force of infection and derive a probability distribution for *R*_*t*_ to accommodate uncertainty in the estimates. Then, from the incidence series and the population at risk, we calculate the attack ratio for each epidemic. We apply our method to estimate *S*_0_ before every major dengue epidemic in the city of Rio de Janeiro, Brazil in the last 18 years.

## Methods

In this section we will start by describing the data and then the method used to estimate the effective reproductive number, *R*_*t*_, from the data and obtain its posterior distribution. We then proceed to describe the Susceptible-Infectious-Recovered (SIR) model used to represent the aggregated disease incidence and how *R*_*t*_ can be integrated into the model to allow for time varying force of infection. Next, an approach to approximate the posterior distributions of the numbers of susceptible to the main circulating dengue viruses for each epidemic is detailed. Finally, we discuss how to estimate the attack ratio of each epidemic using the estimated susceptible fraction and the observed incidence.

### Data

The data used in this paper consists of time series of weekly notified cases of dengue for the city of Rio de Janeiro from 1996 to 2014. The cases are notified based only on clinical criteria. Laboratory confirmation and serotype information are available only for a very small sample and only on recent years (2010–2013). For the parameter estimation procedures incidence was normalized by dividing the number of cases reported by the total city population at each year as given by the census (Census Bureau, Brazilian Institute of Geography and Statistics, http://www.ibge.gov.br/english/).

### Estimating the effective reproductive number (*R*_*t*_)

In monitoring of infectious diseases, it is important to assess whether the incidence of a particular disease is increasing significantly, in order to decide to take preventive measures. The effective reproductive number at time *t*, *R*_*t*_, can be understood as a real-time estimate of the basic reproductive number (*R*_0_) and is defined as the average number of secondary cases per primary case at time *t*.

Let *Y*_*t*_ be the number of reported disease cases for a particular time *t* ∈ (0, *T*). Nishiura el al. (2010)^16^ extend the theory developed by Stallybrass *et al.* (1931)^18^ and propose to estimate *R*_*t*_ as


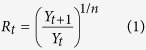


where *n* is taken to be the ratio between the length of reporting interval and the mean generation time of the disease. Here we are interested in the simpler case *n* = 1. If *R*_*t*_ is to be used as a decision tool, however, one needs to be able to quantify the uncertainty about estimate in equation 1. Here we detail how to obtain credibility intervals for *R*_*t*_ under the assumption that the counts *Y*_*t*_ are Poisson distributed for all *t*.

We explore the approach of Ederer and Mantel^19^, whose objective was to obtain confidence intervals for the ratio of two Poisson counts. Let *Y*_*t*_ ~ *Poisson*(*λ*_*t*_) and *Y*_*t*+1_ ~ *Poisson*(*λ*_*t*+1_) and define *W* = *Y*_*t*_ + *Y*_*t*+1_. The authors note that by conditioning on the sum *W*


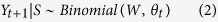



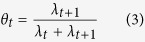


Let 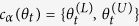 be such that 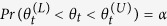. Analogously, define 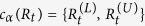 such that 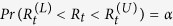. Ederer and Mantel (1974)^19^ show that one can construct a 100*α*% confidence interval for *R*_*t*_ by noting that


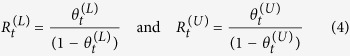


Because the transform from *θ* to *R*_*t*_ is monotonically increasing, the result holds for confidence and credibility intervals alike.

Many authors have chosen to quantify the uncertainty about *θ* following orthodox approaches (see for example^20^,^21^) mainly for simplicity. We choose instead to take a Bayesian approach and use the 100*α*% posterior credibility interval for *θ*_*t*_ as *c*_*α*_(*θ*_*t*_). If we choose the conjugate beta prior with parameters *a*_0_ and *b*_0_ for the binomial likelihood in (2), the posterior distribution for *θ*_*t*_ is


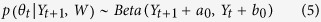


Combining equations (4) and (5) tells us that the induced posterior distribution of *R*_*t*_ is a beta prime (or inverted beta) with parameters *a*_1_ = *Y*_*t*+1_ + *a*_0_ and *b*_1_ = *Y*_*t*_ + *b*_0_^22^. The density of the induced distribution is then


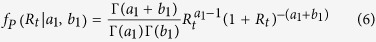


Thus, the expectation of *R*_*t*_ is *a*_1_/(*b*_1_ − 1) and its variance is *a*_1_(*a*_1_ + *b*_1_ − 1)/((*b*_1_ − 2)(*b*_1_ − 1)^2^). Note that this result holds only for *n* = 1. Sampling from the posterior in (6) can be made straightforward by first sampling from (5) and then applying the transform in (4). Also, one can choose *a*_0_ and *b*_0_ so as to elicit meaningful prior distributions for *R*_*t*_. We show how to elicit the prior for *R*_*t*_ from specified prior mean and variance or coefficient of variation in the Appendix.

Also, since *R*_*t*_ > 1 indicates sustained transmission, one may be interested in computing the probability of this event. This can be easily achieved by integrating (6) over the appropriate interval. By noting that


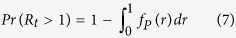



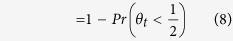


one can compute the desired probability while avoiding dealing with the density in (6) directly.

### Mathematical modelling

A Susceptible-Infectious-Removed (SIR) model is proposed to model dengue dynamics. In the traditional formulation of the model, transmission is governed by a constant transmission rate *β* and recovery happens at a rate *τ*.

For our analysis we chose to let the force of infection vary with time, just as it does in the actual epidemics, as seen in the data. So as the epidemic progresses, the effective transmission rate changes and is given by


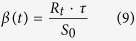


where *R*_*t*_ is the effective reproductive number, estimated as in 1 and *S*_0_ is the initial fraction of susceptible individuals. The complete model with the time-varying force of infection is given by the system of ordinary differential equations:


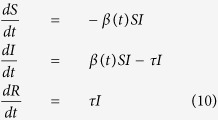


where *S* + *I* + *R* = 1 ∀ *t*. Of course, this is a rather simplified model, in which, for instance, the vector is omitted. The rationale for this simplification is based on the ability of the empirically derived *R*_*t*_ to incorporate the effects of the fluctuating vector populations. Although demography is not included in the model – population is assumed to remain constant throughout an epidemic – the population variation from year to year is taken into consideration as the prevalences are calculated for each year by dividing the number of cases by the official population estimated by the census. In Brazil, dengue affects all age groups which still haven’t been exposed to all 4 serotypes. This is in contrast with countries in which the four serotypes have been endemic for a very long period of time, where dengue mostly affect the youth.

Also, although there are multiple circulating serotypes, our approach can not discriminate between them due to the lack of serotype-specific data. Nevertheless, this modelling strategy can still provide some insight into the disease dynamics and allows us to estimate the initial fraction of susceptibles *S*_0_, a key epidemiological parameter.

### Bayesian parameter estimation

We take a Bayesian approach to the estimation of *S*_0_. First the incidence time series was divided into *J* = 13 epidemic windows that corresponded to significant raises in incidence and normalized to lie on the [0, 1] interval. For a given interval 
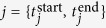
 we observe an incidence time series **Y**_**j**_. We are thus interested in the posterior distribution


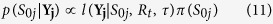


The likelihood *l*(**Y**_**j**_| ⋅) is a approximated by a Normal distribution with fixed variance *σ*^2^. This approximation is a numerical convenience since it confers better stability to the MCMC sampling. In this estimation procedure we kept *R*_*t*_ fixed at fixed at the posterior mean obtained as described above and fixed *τ* = 1/7days^−1^. To complete the inference, we need to specify prior distributions for the parameters of interest. We place a flat Beta(1, 1) prior on *S*_0*j*_ ∀ *j*

To approximate the posterior in (11) we use Markov chain Monte Carlo techniques implemented in the Bayesian inference with Python (BIP)^17^ available at http://code.google.com/p/bayesian-inference/. BIP uses a Differential Evolution Adaptive Metropolis (DREAM)^23^ scheme that efficiently samples from high-dimensional joint distributions using multiple adaptive chains running in parallel with delayed rejection (see Appendix for details). Also, as the numerical integration routine implemented within BIP needs *β*(*t*) to be available at arbitrary values of *t*, i.e., as continuous function of time because of the variable step size, we used linear interpolation to obtain values of *R*_*t*_ for any time point. In this study we used one chain per parameter, i.e, 3 chains for each run. The chains were run until 5000 samples were obtained after discarding 500 burn-in samples. Convergence of the parallel chains was verified at every 100 iterations by the calculation of the Gelman-Rubin’s R (potential scale reduction factor), which approaches 1 at convergence^24^.

### Calculating the attack ratio

The attack ratio of an epidemic is defined by the number of infections divided by the size of the population at risk.


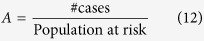


Based on what has been discussed so far, we can rewrite (12) for each epidemic *j* as


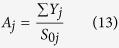


where *S*_0*j*_ is the number of susceptibles before each epidemic *j*, which we estimated before.

Python and R code to perform all the analyses described above is publicly available at https://github.com/fccoelho/paperLM1.

## Results and Discussion

In this paper we propose a method to bypass the lack of serotype-specific case data by informing the time-varying force of infection with the instantaneous reproductive number, *R*_*t*_ which we calculate from aggregated data. The main contribution of this paper can be summarized in the following items: (i) we develop a method to quantify uncertainty about *R*_*t*_ that is readily applicable to other diseases and; (ii) *R*_*t*_ is used to inform a dynamic epidemic model with time-varying force of infection in order to gain insight into the attack ratio of each epidemic; (iii) we propose an estimation procedure for circulating serotype’s *S*_0_ from aggregate case data, which is robust to epidemic sizes; (iv) AR estimates are provided for 18 years of Dengue epidemics in Rio de Janeiro, Brazil.

**Figure 1:**
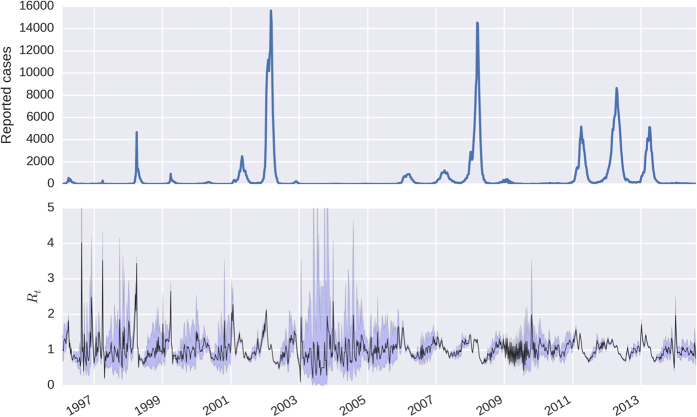
Estimated time-series for *R*_*t*_, along with 95% credible intervals. Top panel show reported cases from which *R*_*t*_ is estimated. As expected, uncertainty about *R*_*t*_ is greater when the case counts are low, for instance in the period 2003–2006, which represented a big hiatus between major epidemics. The intrinsic variability of *R*_*t*_ can be used to inform the time-varying force of infection, since it reflects variation in the vector population and other environmental factors such as temperature and seasonal variation.

Figure 1 shows the *R*_*t*_ series, according to Equation 1 16 along with the confidence bands derived in this paper. It can be seen that the inter-epidemic periods are characterized by *R*_*t*_ being indistinguishable from 1. Due to the intrinsic variability of the *R*_*t*_ series, the examination of its credible intervals is essential to identify periods of sustained transmission. The wider intervals between epidemics are due to the scarcity of cases during these periods. credibility intervals, and therefore offers protection against false alarms (see the section on tail behaviour in the Appendix for a detailed explanation).

A key epidemiological quantity is the attack ratio (AR) of an epidemic, a measure of morbidity and speed of spread which can be used to predict epidemic size and help efficient Public Health planning. The AR depends fundamentally on the population at risk, which in the case of dengue is every naive (to a particular serotype) individual in the population. Estimating the initial susceptible fraction *S*_0_ for each epidemic is thus central to the estimation of the AR. Methods for estimating the number of susceptibles have been proposed before, for other diseases^10^,^11^. These methods attempt to reconstruct the entire series of infectious and susceptibles for measles outbreaks from case data. In the case of dengue, the full (multi-year/multi-epidemic) series of susceptibles to all possible serotypes, cannot be reconstructed based solely on a deterministic transmission model, since the arrival/re-emergence of new serotypes (which are a stochastic events) can change drastically the pool of susceptibles throwing off any sequential estimation based on the incidence dynamics.

Since there is very limited information regarding the actual proportions of each virus in circulation and most information available is about the predominant serotypes for some epidemics in the period of study only^25^, we propose the use of a simplified a single strain model. The main argument we put forward is that by conditioning on the *R*_*t*_ series, we implicitly take into account the variability introduced by the co-circulation of multiple serotypes and heterogeneous levels of immunity in the general population. We sought to deal with all important sources of uncertainty impinging on the estimation of the AR of a dengue epidemic, but not all could be satisfactorily addressed in this analysis. For instance, in any given epidemic there is a large number of mild and asymptomatic cases, which nevertheless acquire immunity. It is estimated that for every case reported, up to 10–20 are not seen by health authorities^26^. Another source of uncertainty is under-reporting of diagnosed cases, which is a serious issue in the health care systems of many developing countries such as Brazil. Duarte and França (2006)^27^, estimated the sensitivity of Dengue reporting for hospitalized patients in Belo-Horizonte, Brazil to be of 63%, meaning that approximately 37% of the suspected Dengue cases go unreported. Lastly, demography and migrations affect the number of susceptible in ways which are not easy to fully determine.

**Figure 2:**
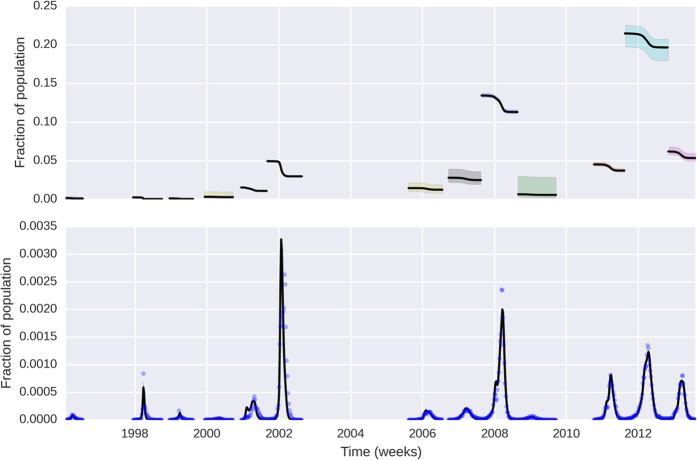
Susceptibles and Infectious posterior curves. The curves were estimated only for the periods where *R*_*t*_ > 1. The susceptible curves in the top panel reflect the prevalence of fraction of susceptibles to circulating strain(s) for each epidemic/outbreak. In the lower panel, we see the posterior distribution of infectious curves, represented by its median and 95% credible interval. Credible intervals are very narrow, and can be hard to distinguish from the median line. Dots show the observed cases, scaled as fractions of the entire population.

**Figure 3:**
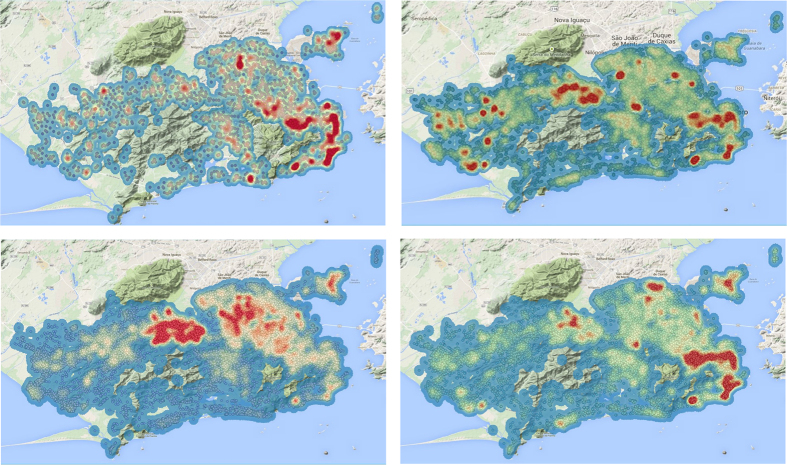
Maps showing the incidence of dengue in the city of Rio de Janeiro from 2010 to 2013–Clockwise from top left. Circles indicate individual notified cases. A heatmap is overlayed on the maps showing absolute density of cases. It can be seen that several areas of the city were affected and no region seems to be free of transmission risk. This suggests that although transmission risk varies spatially, there is significant exposure over the entire city. Maps were generated with QGIS GIS software, from incidence data.

Figure 2, shows the model from (10) fitted to the data. Despite its limitations, our simplified model fits the data well. In it we can see that the susceptibles series in each epidemic starts at the estimated level of *S*_0_. The proportion of susceptibles may seem low, but we must remember that these estimates are being affected by an unknown under-reporting factor, which experts suggest is somewhere between 5 and 10, i.e. for every case observed there are 5 or 10 unobserved. Since this under-reporting affects both the numerator and denominator of (13), its effects should cancel out, giving us an unbiased attack ratio estimate. One other possible source of bias which would lead to the underestimation of *S*_0_ could come from a significant part of the population not being exposed to the disease. However, as we can see in Fig. 3, despite the differences in intensity (incidence), the entire city seems to be at risk, with no particularly “protected” areas, at least in the last four epidemics.

Table 1 contains the attack ratios and medians of the *S*_0_ estimated for each epidemic/outbreak. Underreporting of cases, which is known to exist but of which exact figures cannot be determined, will lead to underestimation *S*_0_. However, the attack ratio shall remain unbiased as the underreporting affects both the numerator and the denominator of equation 13. It is interesting to notice that the larger epidemics, in terms of peak size are not the one with the greater attack ratios. This stresses the importance of knowing the immunological structure of the population. Knowing the *S*_0_ for the circulating viruses we can order to more accurately assess the potential impact of a coming epidemic, since particularly virulent types, can be rendered less of a threat by a low *S*_0_. Honório *et al.*^28^ conducted a serological survey in three separate localities within the city, right before the 2008 epidemic, the authors report seroprevalences varying from 56–77.4% which is compatible with our prediction of 87.5% (1 − *S*_0,2007_) for the entire city, considering that we underestimate *S*_0_ due to the underreporting of cases.

We hope that the results presented in paper will motivate public health authorities to invest in annual serological surveys, to determine the susceptibility profile to each dengue virus as well as to estimate the under-reporting factor of the notification system.

## Additional Information

**How to cite this article**: Coelho, F. C. and Carvalho, L. M. d. Estimating the Attack Ratio of Dengue Epidemics under Time-varying Force of Infection using Aggregated Notification Data. *Sci. Rep.* **5**, 18455; doi: 10.1038/srep18455 (2015).

## Supplementary Material

Supplementary Information

## Figures and Tables

**Table 1:** Median attack ratio and 95% credibility intervals calculated according to (13).

^†^Year	^‡^Cases	median Attack Ratio	^‡^*S*_0_	Circulating Serotypes
1996	0.066	0.39 (0.17–0.54)	0.171 (0.12–0.38)	DEN-1 and 2^13^
1997	0.238	0.87 (0.74–0.87)	0.273 (0.27–0.32)	DEN-1 and 2^13^
1998	0.0708	0.5 (0.49–0.5)	0.142 (0.14–0.14)	DEN-1 and 2^13^
1999	0.0371	0.11 (0.037–0.2)	0.345 (0.18–1.0)	DEN-1, 2 and 3^14^
2000	0.394	0.25 (0.24–0.27)	1.55 (1.5–1.6)	DEN-1, 2 and 3^14^
2001	2.38	0.48 (0.47–0.49)	4.95 (4.8–5.1)	DEN-1, 2 and 3^14^
2005	0.217	0.15 (0.1–0.21)	1.47 (1.0–2.1)	
2006	0.315	0.11 (0.08–0.14)	2.81 (2.2–3.7)	DEN-2^15^
2007	2.03	0.15 (0.15–0.15)	13.5 (13.0–14.01)	DEN-2^15^
2008	0.0923	0.14 (0.031–0.31)	0.672 (0.3–2.4)	DEN-2^15^
2010	0.831	0.18 (0.17–0.19)	4.54 (4.3–4.8)	DEN-2^15^
2011	1.85	0.086 (0.082–0.094)	21.5 (20.0–23.0)	DEN-1, 2 and 4^15^
2012	0.864	0.14 (0.13–0.15)	6.21 (5.8–6.8)	

Values are presented as percentage of total population. ^†^Year corresponds to the start of the epidemic, but the peak of cases may occur in the following year. ^‡^percentage of population. These results show considerable variation in AR between epidemics, consistent with the accquiring and loss of serotype-specific immunity.
