# Supplementary material for: Estimating the Attack Ratio of Dengue Epidemics under Time-varying Force of Infection using Aggregated Notification Data
Source: Sci Rep. 2015 Dec 17;5:18455. doi: 10.1038/srep18455 (PMC4682072; doi:10.1038/srep18455)
Supplement: Supplementary Information [file srep18455-s1.pdf]

Supplementary material to "Estimating the Attack Ratio of Dengue Epidemics under Time-varying Force of Infection using Aggregated Notification Data"

Flavio Codeço Coelho and Luiz Max de Carvalho

August 3, 2015

A remark on prior distributions and tail behaviour of the distribution of R_t

There are a number of approaches to deriving the distribution of R_t . Alternatively to the approach described in the main text [1], one could use the conditional distribution of R_t on Y_{t+1} and Y_t as defined in equation A7 of Nishiura et al. [2]:

$$f_R(R_t) = (Y_t R_t)^{Y_{t+1}} e^{-Y_t R_t} \tag{1}$$

Noticing the kernel of (1) is that of a gamma distribution with $a_2 = Y_{t+1} + 1$ and $b_2 = Y_t$, we obtain a proper density from which to construct $c_\alpha(R_t)$, simply by computing the appropriate quantiles of said distribution. This density is

$$f_N(R_t|a_2, b_2) = \frac{b_2^{a_2}}{\Gamma(a_2)} R_t^{a_2-1} e^{-b_2 R_t} \tag{2}$$

In order to decide which approach to take, it may be of use analysing the tail behaviour of the derived distributions for R_t . Consider the case of using a flat Uniform$(0, 1)$ prior for θ_t. With $a_0 = b_0 = 1$, $a_1 = a_2$ and $b_1 = b_2 + 1$. The beta prime (inverse beta distribution) will have heavier tails compared to the conditional distribution proposed by [2], thus providing more conservative confidence/credibility intervals. To see that one needs simply take the ratio of the Beta prime and Gamma (unnormalized) densities and evaluate the limit as R_t goes to infinity:

$$\lim_{R_t\to\infty} \frac{f_P(R_t|a_1, b_1)}{f_N(R_t|a_2, b_2)} = \lim_{R_t\to\infty} \frac{e^{Y_t R_t}}{(1 + R_t)^{Y_t+Y_{t+1}+2}} = \infty \tag{3}$$

Note also that we deliberately construct $c_\alpha(R_t)$ as a equal-tailed $100\alpha\%$ credible set, rather than a less conservative highest posterior density (HPD) interval. Finally, we performed a simple simulation study to assess the result in equation 3. The simulations were carried out as follows:

1. Create a two-dimensional grid of values for λ_1 and λ_2, with rate values from 2 to 1000;

2. For each point $(\lambda_1^{(j)}, \lambda_2^{(j)})$ in the grid,

   - Generate 1000 realisations of the random variables $Y_1^{(ij)} \sim \text{Poisson}(\lambda_1^{(j)})$ and $Y_2^{(ij)} \sim \text{Poisson}(\lambda_2^{(j)})$, with $i = 1, 2, \ldots, 1000$ and compute $R_t^{(ij)} = Y_2^{(ij)}/Y_1^{(ij)}$;
   - Compute the α% credibility/confidence intervals using the method proposed here (β) and using the distribution proposed by Nishiura (2010) [2] (Γ), denoted by $c_\beta(R_t^{(ij)}; \alpha)$ and $c_\Gamma(R_t^{(ij)}; \alpha)$ respectively;
   - Determine whether
     (i) $c_\Gamma(R_t^{(ij)}; \alpha) \subseteq c_\beta(R_t^{(ij)}; \alpha)$;
     (ii) $c_\beta(R_t^{(ij)}; \alpha) \subseteq c_\Gamma(R_t^{(ij)}; \alpha)$ or;
     (iii) there is overlap
   - Calculate $Pr(R_t^{(ij)} > 1)$ using both distributions

The results show that the credibility intervals obtained using the distribution proposed here were larger than (i.e. contained) those computed using the distribution in 2 in 90.69% of the simulations (integrating over the grid), while the converse was observed in 1.14% of the cases. Moreover, our method yielded lower $Pr(R_t > 1)$ in 79.48% of the simulations, indicating it is indeed more robust to false alarm. An R script to perform the simulation study described above can be found at `https://github.com/fccoelho/paperLM1/blob/master/R/credibility_comparison.R`.

As a side note, the Bayesian approach presented in this paper will give similar results to orthodox confidence intervals [3] and [4] for Y_{t+1} and $Y_t >> 1$. Under the flat uniform prior for θ_t, the Bayesian posterior credibility interval is nearly indistinguishable from the confidence interval proposed by Clopper & Pearson (1931) [4] for $Y_{t+1}, Y_t > 20$. Note also that the uniform prior ($Beta(1, 1)$) for θ_t constitutes a poor prior choice mainly because the induced distribution for R_t is only well-defined for $b_0 > 2$.

An advantage of the Bayesian approach is that one can devise prior distributions for θ_t taking advantage of the intuitive parametrization and flexibility of the beta family of distributions. Prior elicitation can also be done for R_t and the hyper-parameters directly plugged into the prior for θ_t. One can, for example, choose prior mean and variance for R_t and find a_0 and b_0 that satisfy those conditions. Let m_0 and v_0 be the prior expectation and variance for R_t. After some tedious algebra one finds

$$a_0 = \frac{m_0 v_0 + m_0^3 + m_0^2}{v_0} \tag{4}$$

$$b_0 = \frac{2v_0 + m_0^2 + m_0}{v_0} \tag{5}$$

If one wants only to specify m_0 and the coefficient of variation $c = \sqrt{v_0}/m_0$ for R_t *a priori*, some less boring algebra gives:

$$a_0 = \frac{m_0^3 c^2 + m_0^3 + m_0^2}{m_0^2 c^2} \tag{6}$$

$$b_0 = \frac{2m_0^2 c^2 + m^2 + m}{m_0^2 c^2} \tag{7}$$

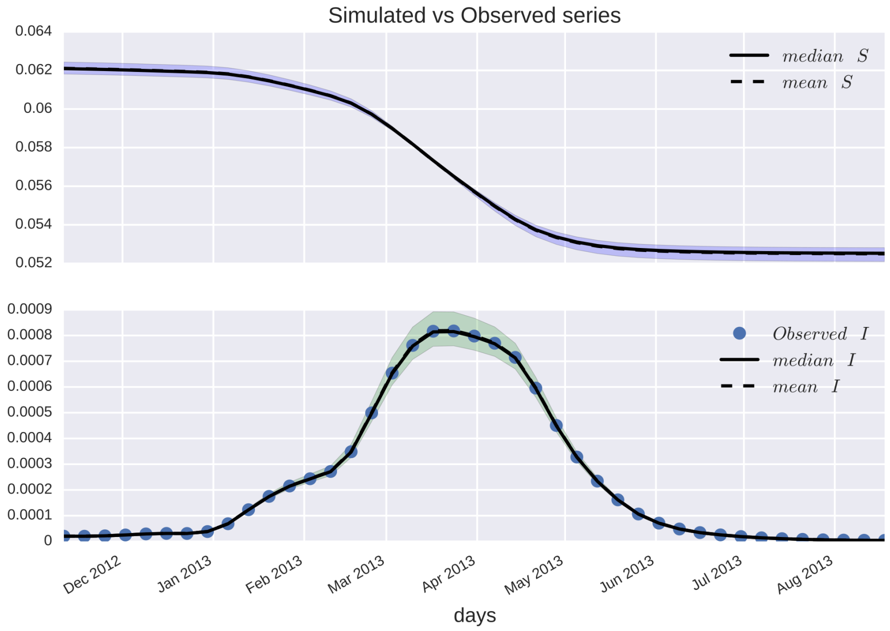

Figure 1: S and I series fitted to simulated incidence data (blue dots) with $S_0 = 0.0621$, and R_t estimated from real incidence data.

This approach thus makes it possible to incorporate epidemiological knowledge about disease Biology (e.g. the magnitude of R_0) into the computation of R_t . This may prove particularly important when disease counts are low and/or close to the detection threshold. We provide an R script to perform the elicitation described above at `https://github.com/fccoelho/paperLM1/blob/master/R/elicit_Rt_prior.R`.

Estimating S_0 from simulated data

In order to determine whether the inference methodology proposed can recover the true parameter values of the underlying simulation model, we have devised a simple simulation experiment. Using the SIR model presented in the main paper we have simulated the incidence curve of the 2012-13 epidemic, using $S_0 = 0.0621$ and R_t , as estimated from actual incidence data, and $\tau = 1$. Figure 1 shows the posterior S and I alongside with simulated incidence data. It is clear that the method can recover the correct value for S_0 used to simulate incidence. The script to generate the simulated data and run the inference is available at the paper's Github repository (`https://github.com/fccoelho/paperLM1/blob/master/python/fit_simulated_data.py`).

DiffeRential Evolution Adaptive Metropolis (DREAM)

Since the posterior distributions desired in this paper are not available in closed-form, we need to resort to numerical methods to obtain approximations. We employ an adaptive Markov chain Monte Carlo (MCMC) algorithm, proposed by [5], called DiffeRential Evolution Adaptive Metropolis (DREAM).

DREAM draws on the basic structure of Differential Evolution Markov Chain (DE-MC) which runs N independent chains in parallel and accepts moves proportional to the difference of two randomly sampled members (chains), thus differentially evolving conditional on the proposal variances. An additional aspect of DREAM is the so-called delayed rejection. This procedure adapts the original Metropolis-Hastings algorithm by attempting new moves whenever a proposal is rejected (instead of setting the current state to the previously accepted one), thus delaying the rejection of proposals. This potentially decreases autocorrelation and improves mixing (see [6] for details).

A desirable property of DREAM is that one can scale the algorithm with model complexity, meaning one can initialise as many parallel chains as there are parameters in the model, thus exploiting the multi-core architecture of modern computers to speed up convergence of the MCMC.

A Python implementation of DREAM for dynamic models is available in the package Bayesian inference with Python (BIP) [7] available at `http://code.google.com/p/bayesian-inference/`. The code to produce the results presented in this paper is available at `https://github.com/fccoelho/paperLM1/tree/master/python`.

References

[1] Ederer, F. & Mantel, N. Confidence limits on the ratio of two poisson variables. *American Journal of Epidemiology* **100**, 165–167 (1974).

[2] Nishiura, H., Chowell, G., Heesterbeek, H. & Wallinga, J. The ideal reporting interval for an epidemic to objectively interpret the epidemiological time course. *J R Soc Interface* **7**, 297–307 (2010).

[3] Wilson, E. B. Probable inference, the law of succession, and statistical inference. *Journal of the American Statistical Association* **22**, 209–212 (1927).

[4] Clopper, C. & Pearson, E. S. The use of confidence or fiducial limits illustrated in the case of the binomial. *Biometrika* 404–413 (1934).

[5] Vrugt, J. A. *et al.* Accelerating markov chain monte carlo simulation by differential evolution with self-adaptive randomized subspace sampling. *International Journal of Nonlinear Sciences and Numerical Simulation* **10**, 271–288 (2008).

[6] Mira, A. On metropolis-hastings algorithms with delayed rejection. *Metron* **59**, 231–241 (2001).

[7] Coelho, F. C., Codeço, C. T. & Gomes, M. G. A Bayesian framework for parameter estimation in dynamical models. *PLoS ONE* **6**, e19616 (2011).
